# Long-lasting memory deficits in mice withdrawn from cocaine are concomitant with neuroadaptations in hippocampal basal activity, GABAergic interneurons and adult neurogenesis

**DOI:** 10.1242/dmm.026682

**Published:** 2017-03-01

**Authors:** David Ladrón de Guevara-Miranda, Carmelo Millón, Cristina Rosell-Valle, Mercedes Pérez-Fernández, Michele Missiroli, Antonia Serrano, Francisco J. Pavón, Fernando Rodríguez de Fonseca, Magdalena Martínez-Losa, Manuel Álvarez-Dolado, Luis J. Santín, Estela Castilla-Ortega

**Affiliations:** 1 Departamento de Psicobiología y Metodología de las Ciencias del Comportamiento, Instituto de Investigación Biomédica de Málaga (IBIMA), Facultad de Psicología, Universidad de Málaga, 29071 Málaga, Spain; 2 Departamento de Fisiología, Instituto de Investigación Biomédica de Málaga (IBIMA), Facultad de Medicina, Universidad de Málaga, 29071 Málaga, Spain; 3 Laboratory of Cell-based Therapy for Neuropathologies, Centro Andaluz de Biología Molecular y Medicina Regenerativa (CABIMER), 41092 Sevilla, Spain; 4 Unidad de Gestión Clínica de Salud Mental, Instituto de Investigación Biomédica de Málaga (IBIMA), Hospital Regional Universitario de Málaga, 29010 Málaga, Spain

**Keywords:** Anxiety, c-Fos, Parvalbumin, Neuropeptide Y, Cell proliferation, Behavior-induced neuroplasticity

## Abstract

Cocaine addiction disorder is notably aggravated by concomitant cognitive and emotional pathology that impedes recovery. We studied whether a persistent cognitive/emotional dysregulation in mice withdrawn from cocaine holds a neurobiological correlate within the hippocampus, a limbic region with a key role in anxiety and memory but that has been scarcely investigated in cocaine addiction research. Mice were submitted to a chronic cocaine (20 mg/kg/day for 12 days) or vehicle treatment followed by 44 drug-free days. Some mice were then assessed on a battery of emotional (elevated plus-maze, light/dark box, open field, forced swimming) and cognitive (object and place recognition memory, cocaine-induced conditioned place preference, continuous spontaneous alternation) behavioral tests, while other mice remained in their home cage. Relevant hippocampal features [basal c-Fos activity, GABA^+^, parvalbumin (PV)^+^ and neuropeptide Y (NPY)^+^ interneurons and adult neurogenesis (cell proliferation and immature neurons)] were immunohistochemically assessed 73 days after the chronic cocaine or vehicle protocol. The cocaine-withdrawn mice showed no remarkable exploratory or emotional alterations but were consistently impaired in all the cognitive tasks. All the cocaine-withdrawn groups, independent of whether they were submitted to behavioral assessment or not, showed enhanced basal c-Fos expression and an increased number of GABA^+^ cells in the dentate gyrus. Moreover, the cocaine-withdrawn mice previously submitted to behavioral training displayed a blunted experience-dependent regulation of PV^+^ and NPY^+^ neurons in the dentate gyrus, and neurogenesis in the hippocampus. Results highlight the importance of hippocampal neuroplasticity for the ingrained cognitive deficits present during chronic cocaine withdrawal.

## Abstract (precis)

**Summary:** Hippocampal functional activity and neuroplasticity are altered in mice long withdrawn from cocaine, with concomitant memory deficits, emphasizing the role of the hippocampus in persistent cognitive manifestation of cocaine withdrawal.

## INTRODUCTION

The use of illicit drugs is one of the most serious health problems in the western world, and cocaine is the most widely used psychostimulant drug (EMCDDA, 2015; UNODC, 2015). Cocaine abuse usually carries an important socioeconomic burden, with both physical and mental health dysfunctions, including the engagement in a cocaine addiction disorder when casual users become dependent on this substance (Lopez-Quintero et al., 2011). A growing body of literature supports the premise that persistent emotional and cognitive symptoms contribute to the chronic and relapsing nature of cocaine addiction. At the emotional level, cocaine withdrawal is often associated with negative effects involving a high-anxiety state and intense desire for the drug (‘craving’) that urges the individual to resume drug intake (Koob and Zorrilla, 2010). Moreover, there is an elevated incidence of mood and anxiety disorders among cocaine addicts (Araos et al., 2015, 2014; Pedraz et al., 2015a,b) and such psychiatric comorbidity yields a worse prognosis (Siqueland et al., 2002). Cocaine-dependent individuals also show broad cognitive deficits [including attention, working memory, reference memory, behavioral inhibition or cognitive flexibility (Spronk et al., 2013; Vonmoos et al., 2013, 2014)] that are therapeutically relevant as they predict relapse and low levels of treatment retention (Aharonovich et al., 2006; Fox et al., 2009; Teichner et al., 2002). These affective and cognitive symptoms in cocaine addicts are, at least in part, caused by the repeated cocaine use (Vonmoos et al., 2014), and pre-clinical studies have shown that cocaine exposure and withdrawal induce both emotional (Perrine et al., 2008; Sarnyai et al., 1995) and cognitive alterations (Briand et al., 2008; Krueger et al., 2009; Mendez et al., 2008) in rodents.

The hippocampal region is emerging as a strong candidate that significantly contributes to the cocaine-induced emotional and cognitive symptoms (Castilla-Ortega et al., 2016b). This limbic structure has a long established role to regulate anxiety and depression-like responses, as well as key cognitive functions such as working and reference memory (Bannerman et al., 2004; Walf and Frye, 2006). As revealed by post-mortem samples, the hippocampus from cocaine addicts shows a notable dysregulation in the expression of genes involved in glutamatergic and GABAergic transmission (Enoch et al., 2014, 2012), as well as in cellular plasticity and function (Mash et al., 2007; Zhou et al., 2011). Moreover, the hippocampus is integrated into the ‘addiction brain circuit’ along with the main reward areas, establishing reciprocal functional connections that are sculpted by cocaine exposure (Castilla-Ortega et al., 2016b). Resting-state functional neuroimaging studies in cocaine addicts reveal a whole-brain functional connectivity reduction where the hippocampus stands out as one of the brain structures most evidently disconnected from other regions (Castilla-Ortega et al., 2016b; Ding and Lee, 2013). In experimental conditions where the cocaine-dependent individuals are exposed to cocaine-associated cues, hippocampal activity is then greatly increased and correlated with craving (Castilla-Ortega et al., 2016b), whereas increased hippocampal activation in resting conditions predicts the likelihood to relapse in cocaine use (Adinoff et al., 2015). Considering that poorer cognitive performance – affecting hippocampal-dependent functions such as spatial ability and verbal memory – predicts treatment dropout and further cocaine intake in cocaine addicts receiving treatment (Aharonovich et al., 2006; Fox et al., 2009; Teichner et al., 2002), it is possible for the hippocampus to hold a common neurobiological substrate that underlies both the impaired cognition and the relapse outcomes in cocaine addicts, thus being a relevant therapeutic objective. Nevertheless, the hippocampus has received relatively scarce attention in research into cocaine addiction as it is not among the classical addiction-related brain areas (e.g. Everitt, 2014; Everitt et al., 2008).

Hippocampal activity and function are tightly regulated by neuroplastic and neurogenic processes in the dentate gyrus (DG), a region that is the main input for the hippocampus and highly responsive to the environmental demands (Castilla-Ortega et al., 2011). In fact, the DG is one of the few neurogenic niches in the adult brain. Adult hippocampal neurogenesis (AHN) is required for normal hippocampal function, since its ablation results in aberrant emotionality (Revest et al., 2009) and impaired cognition (Castilla-Ortega et al., 2011; Deng et al., 2010; Leuner et al., 2006). In contrast, increased AHN potentiates memory and alleviates emotional dysregulation (Castilla-Ortega et al., 2011; Deng et al., 2010; Leuner et al., 2006). Importantly, the adult-born DG neurons are modulated by a number of stimuli that may upregulate (e.g. hippocampal-dependent learning activities, physical exercise) or reduce (e.g. stressful experiences) their proliferation, maturation and/or survival, with subsequent implications for behavior (Castilla-Ortega et al., 2011; Deng et al., 2010; Leuner et al., 2006). Another key neuron population that modulates hippocampal function are the γ-amino butyric acid (GABA)-ergic interneurons, which constitute ∼10% of all hippocampal neurons, and include multiple inhibitory neuron subtypes according to their neurochemical identity (Freund and Buzsáki, 1996; Vizi and Kiss, 1998). In particular, those expressing parvalbumin (PV) or neuropeptide Y (NPY) have demonstrated a role in both anxiety and memory. Thus, an increment in the number of these interneurons usually correlates with anxiolysis and a boosted cognitive performance, whereas their loss leads to detrimental behavioral effects and is associated with several pathological conditions (PV: Boley et al., 2014; Brady and Mufson, 1997; Fuchs et al., 2007; Korotkova et al., 2010; Megahed et al., 2014; Morellini et al., 2010; Quarta et al., 2015; Zou et al., 2016; NPY: Beck and Pourié, 2013; Christiansen et al., 2014; de Lanerolle et al., 1989; Megahed et al., 2014). These neuronal populations also undergo experience-dependent modulation after exercise, environmental enrichment, stress and learning (Arida et al., 2004; Bjørnebekk et al., 2006, 2007; Czeh et al., 2005; Donato et al., 2013; Filipovic et al., 2013; Iuvone et al., 1996; Nguyen et al., 2013; Sergeyev et al., 2005; Suzuki et al., 2014). Interestingly, the NPY stimulates AHN (Decressac et al., 2011; Howell et al., 2005) and the PV^+^ interneurons are involved in early key phases of the AHN process (Song et al., 2013). Therefore, the AHN- PV- and NPY-expressing neurons are inter-related and significantly contribute to the neuroplasticity that regulates hippocampal function and its adaptability to the environmental demands.

In the field of cocaine research, it is known that rodents with reduced AHN engage in more cocaine-seeking and -taking behaviors (Castilla-Ortega et al., 2016a; Deschaux et al., 2014; Noonan et al., 2010) and that cocaine exposure usually reduces AHN (Blanco-Calvo et al., 2014; Castilla-Ortega et al., 2016b). However, young hippocampal neurons and proliferating cells are apparently normalized shortly after cocaine cessation, which complicates the establishment of a strong link between an impaired AHN and the long-lasting cocaine-induced behavioral symptoms (Castilla-Ortega et al., 2016b; Maćkowiak et al., 2005; Xie et al., 2010; Yamaguchi et al., 2005). Nevertheless, it remains to be investigated whether AHN normally responds to environmental stimuli during cocaine withdrawal. In addition, the potential effect of cocaine on the hippocampal GABAergic interneurons, including the PV^+^ and NPY^+^ populations, has been largely unexplored. The present study aims to investigate whether emotional and cognitive symptoms and their associated hippocampal features persist in mice long withdrawn from cocaine, with special emphasis on the neurogenic DG. The effects of cocaine on basal hippocampal activity (assessed by expression of the early-immediate gene *c-fos*), GABA^+^, PV^+^ and NPY^+^ hippocampal neurons, and AHN (proliferating cells and young DG neurons) were analysed by immunohistochemistry, both in undisturbed mice and in mice previously submitted to behavioral training in a battery of emotional and memory tests. In this way, we investigate how exposing mice to a cognitively demanding environment modulates GABAergic interneurons and AHN in the cocaine-treated mice.

## RESULTS

### Cocaine-withdrawn mice show minimal emotional alterations in the absence of exploratory changes

**Fig. 1.**
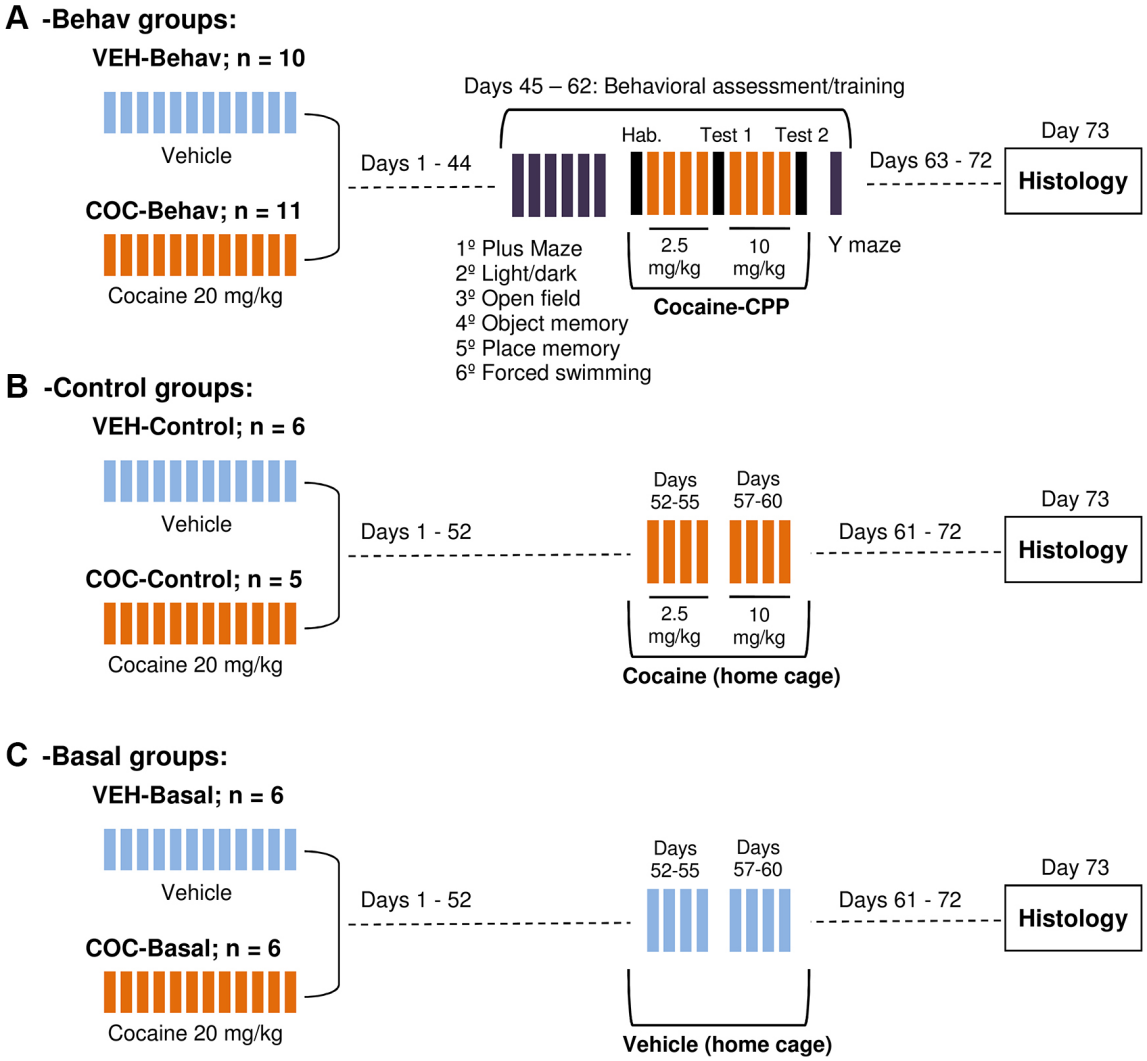
**Experimental protocol.** Dotted lines indicate the periods where mice remained undisturbed in their home cages. (A) The -Behav mice were exposed to chronic cocaine (COC-Behav) or vehicle (VEH-Behav) treatment and were behaviorally assessed 44 days after treatment. All the -Behav mice received cocaine when tested in the CPP apparatus. (B) The -Control mice underwent a cocaine (COC-Control) or vehicle (VEH-Control) treatment similar to the -Behav groups but remained undisturbed in their home cage (no behavioral testing). (C) The -Basal mice received the chronic cocaine (COC-Basal) or vehicle (VEH-Basal) treatment and remained in undisturbed in their home-cage with no further cocaine exposure. CPP, conditioned place preference; Hab., habituation session.

**Fig. 2.**
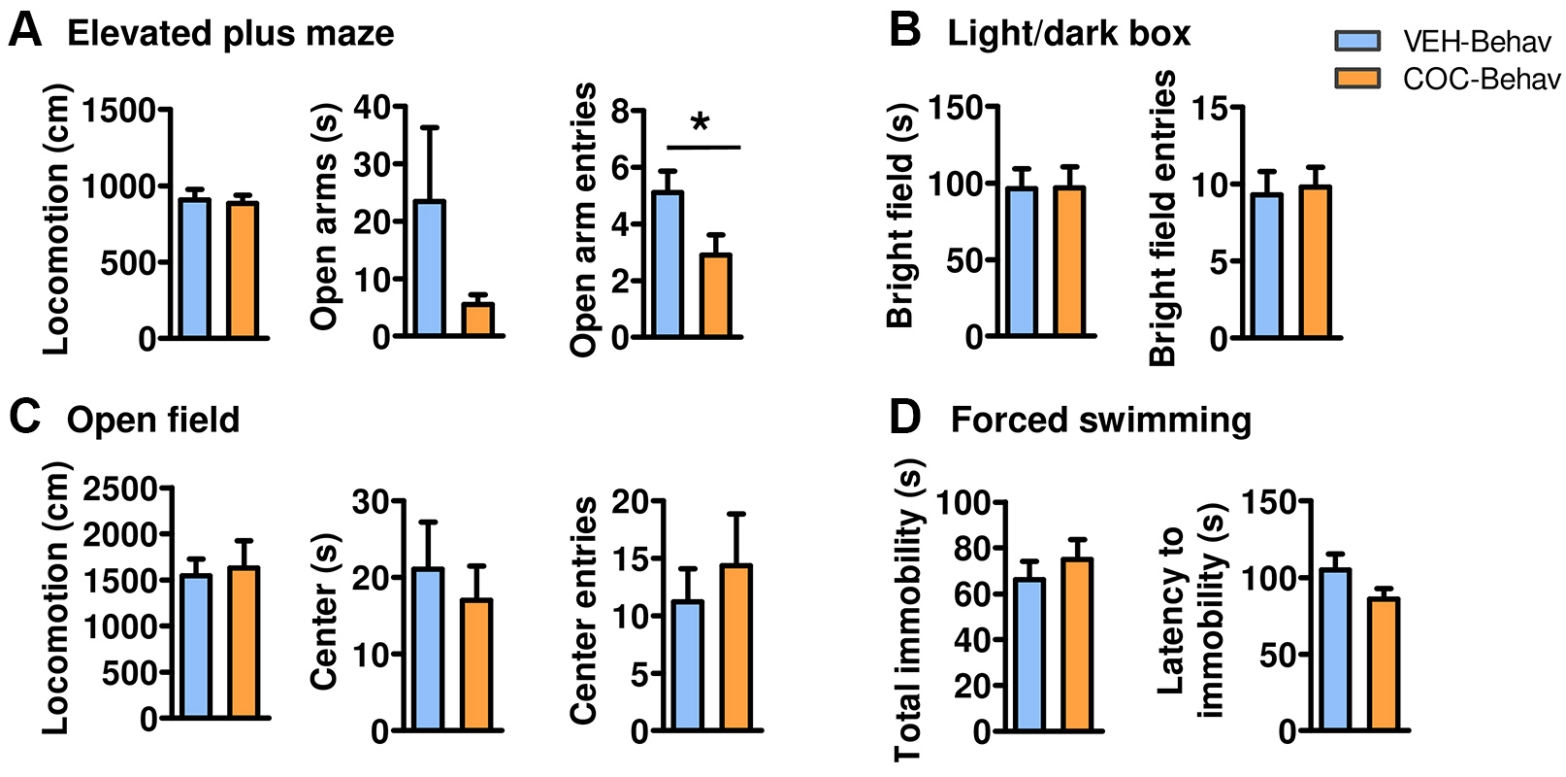
**Cocaine-withdrawn mice showed minimal emotional behavior alteration and no effects on exploratory activity.** The cocaine-treated mice showed a reduced number of open arm entries in the elevated plus maze but no significant differences in the total time in open arms (A). The COC-Behav mice performed normally in the anxiety-like measures evaluated in the light/dark box (B) and in the open field (C) tests, as well as in the forced swimming test for depression-like behavior (D). Locomotion, when evaluated, was unaltered (A,C). Results are represented as means±s.e.m. Student's *t*-tests for VEH versus COC comparisons: *P<0.05.

Behavior was assessed in mice long withdrawn from chronic cocaine treatment (COC-Behav mice) that were compared with vehicle-treated mice (VEH-Behav mice) (Fig. 1A). In order to analyse anxiety-like behavior we performed open field, elevated plus maze and dark/bright-field tests (Fig. 1). Groups were similar in most relevant measures of anxiety in these tests, such as total time spent in the open arms of the elevated plus maze (Fig. 2A), the number of entries and the total time spent in the bright compartment of a light/dark box (Fig. 2B) or exploration of the central area in the open field (Fig. 2C). The only significant change was a reduced number of entries in the plus maze open arms in the COC-Behav mice (*t*_19_=−2.105, *P*=0.049), in the absence of locomotor deficits (Fig. 2A). The forced swimming test did not reveal any differences in depression-like behavior assessed as the latency for first immobility and total immobility time (Fig. 2D), nor in the time the mice struggled in the water (data not shown).

### Chronic cocaine exposure induces persistent cognitive impairment

**Fig. 3.**
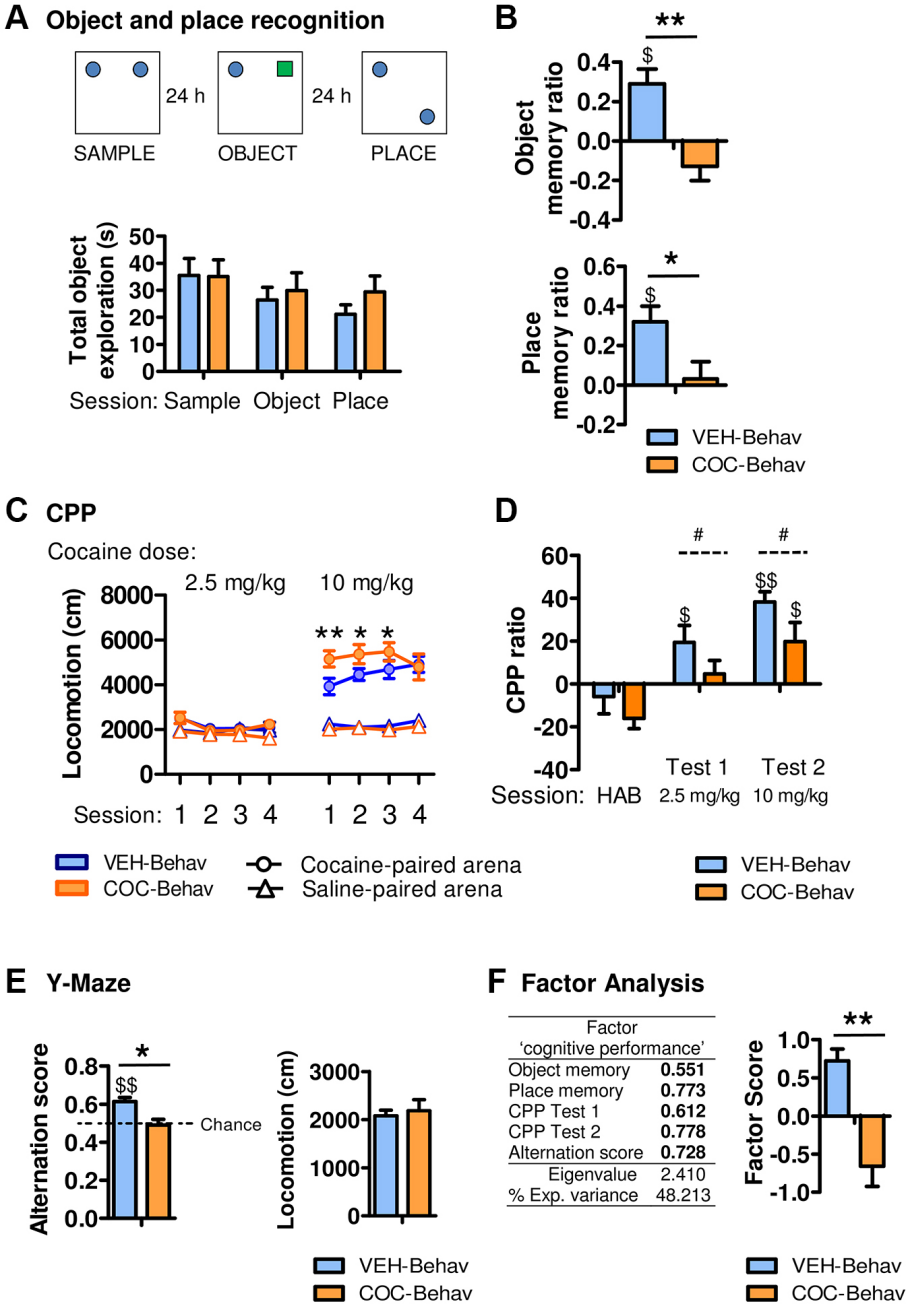
**Cocaine withdrawal induces a persistent impairment in hippocampal-dependent memory and sensitization to its hyperlocomotor effects.** The cocaine-withdrawn mice showed a normal object exploration interval during the recognition sessions (A) but were unable to remember and discriminate the novel or the displaced objects (B). (C) In the cocaine-induced CPP paradigm, the COC-Behav mice showed increased hyperlocomotion when exposed to 10 mg/kg of cocaine, but not in the saline-paired sessions or when challenged with the 2.5 mg/kg cocaine dose, that induced no apparent stimulatory response (HAB, habituation session). (D) Conditioning was blunted in the COC-Behav mice. (E) The COC-Behav mice were also impaired in their Y-maze spontaneous alternation in the absence of locomotor deficits. (F) A factorial analysis that included all memory measures confirmed a worse cognitive performance in the COC-Behav mice. Results are represented as means±s.e.m. Student's *t*-tests for VEH versus COC comparisons: *P<0.05, **P<0.001. One-sample Student's *t*-tests were used to compare means versus zero (B,D) or versus 0.5 (E): ^$^*P*<0.05, ^$$^*P*<0.001 (in B, this comparison indicates a preference for the novel or the displaced object versus the familiar or the static one; in D, it indicates a preference for the cocaine-paired compartment versus the saline-paired one; in E, it indicates a frequency of spontaneous alternation over chance performance). ANOVA treatment effect: ^#^*P*<0.05.

Despite the absence of notable alterations in their emotional and exploratory behavior, the cocaine-withdrawn mice showed a consistent memory deficit. In contrast to the VEH-Behav group, the COC-Behav mice were impaired in discriminating both a novel (*t*_19_=3.933, *P*=0.001) and a displaced object (*t*_19_=2.450, *P*=0.024), performing at chance levels in both tasks (Fig. 3B). It is important to note that mice from both treatment groups spent a similar amount of time exploring objects across trials (Fig. 3A), ruling out a reduced motivation for object exploration.

During conditioned place preference (CPP) training, the locomotor activity induced by cocaine administration was analyzed by a repeated-measures ANOVA [‘treatment’ (VEH or COC)×‘cocaine dose’ (2.5 or 10 mg/kg)×‘compartment’ (saline- or cocaine-paired)×‘session’ (1-4)], which revealed significant effects (‘treatment×compartment’: *F*_1,76_=4.240, *P*=0.043; ‘cocaine dose’: *F*_1,76_=115.940, *P*=0.000; ‘compartment’: *F*_1,76_=120.53, *P*=0.000; ‘dose×compartment’: *F*_1,76_=77.235, *P*=0.000; ‘session×cocaine dose’: *F*_3,228_=5.500, *P*=0.001; ‘session×cocaine dose×compartment’: *F*_3,228_=4.082, *P*=0.008). The *post hoc* comparisons showed an exacerbated locomotor response to the 10 mg/kg cocaine dose in the COC-Behav mice. This hyperlocomotion was most evident in the first exposures since the VEH-Behav mice were progressively sensitized to the locomotor effects of the drug [Fisher's least significant difference (LSD) is shown in Fig. 3C]. However, both groups showed similar locomotion after saline administration or after the 2.5 mg/kg cocaine dose, which was insufficient to induce any stimulating effects. The two groups were similar in the habituation session (*P*>0.05) in the cocaine-induced conditioned response, but a repeated-measures ANOVA across the test sessions revealed worse conditioning in the COC-Behav mice (‘treatment’: *F*_1,19_=4.736, *P*=0.042; ‘session’: *F*_1,19_=7.734, *P*=0.012; Fig. 3D).

Spatial working memory was further evaluated in a Y-maze. The COC-Behav mice performed at chance levels and achieved fewer correct spontaneous alternations than the VEH-Behav mice (*t*_19_=−3.283, *P*=0.004), while no effects were found in locomotion (Fig. 3E). Lastly, a factor analysis (supplementary Materials and Methods) extracted one unique factor in which all the memory-related measures were included (Fig. 3F), representing the mice's cognitive performance. Comparison of the factor scores revealed a better cognitive performance in the VEH-Behav group (*t*_19_=4.359, *P*=0.000; Fig. 3F).

### Withdrawal from chronic cocaine induces a persistently increased DG basal activity

Mice in the behaviorally tested (-Behav) and undisturbed control (-Control) conditions (Fig. 1B) were compared by a repeated-measures ANOVA to assess the impact of chronic cocaine withdrawal and behavioral training on the histological parameters of both the supragranular and infragranular cell layers of the DG [‘treatment’ (VEH or COC)×‘behavior’ (-Control or -Behav)×‘DG blade’ (SupraG or InfraG)].

**Fig. 4.**
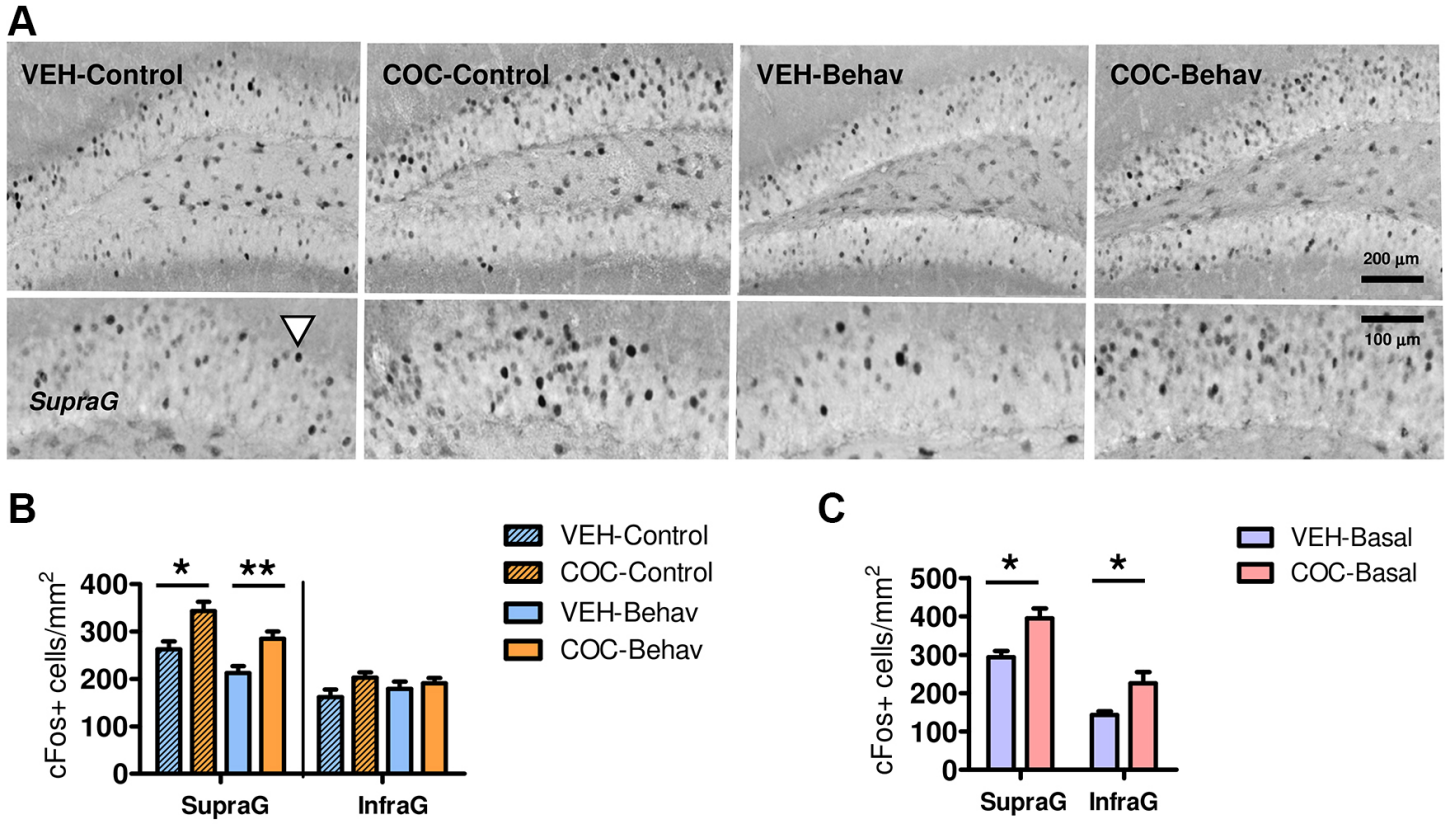
**Increased basal c-Fos expression in the DG of mice withdrawn from cocaine.** (A,B) The cocaine-withdrawn mice showed increased basal c-Fos activity in the supragranular cell layer of the DG, irrespective of their behavioral testing status (-Control or -Behav condition). Arrowhead indicates a positive cell. (C) Increased basal c-Fos activity in the DG was also found in the -Basal mice that were not re-exposed to cocaine nor submitted to behavior testing. Results are represented as the mean±s.e.m. number of positive cells per mm^2^. *Post hoc* LSD test: *P<0.05, **P<0.001, for VEH versus COC.

Basal hippocampal c-Fos expression was evaluated since ‘resting’ brain activity and functional connectivity are profoundly altered in the hippocampus of cocaine addicts (Adinoff et al., 2015; Ding and Lee, 2013). Both the COC-Control and the COC-Behav mice displayed increased basal c-Fos expression in the supragranular region compared with their respective VEH groups (Fig. 4A,B) (‘treatment’: *F*_1,28_=10.899, *P*=0.003; ‘DG blade’: *F*_1,28_=172.39, *P*=0.000; ‘treatment×DG blade’: *F*_1,28_=12.776, *P*=0.001; LSD is shown in Fig. 4B) and there was also an effect of behavioral training in reducing basal c-Fos activity in the supragranular region (‘DG blade×behavior’: *F*_1,28_=16.594, *P*=0.000; Fig. 4A; and analysis in Fig. 7A). Interestingly, increased c-Fos expression in the DG was also found in the COC-Basal mice (‘treatment’: *F*_1,10_=10.235, *P*=0.010; ‘DG blade’: *F*_1,10_=261.31, *P*=0.000; LSD is shown in Fig. 4C). The results revealed persistently increased basal c-Fos expression in the DG of cocaine-withdrawn mice, irrespective of their behavioral treatment. Interestingly, this feature seemed limited to the DG, since further exploration of this marker (carried out in the VEH-Behav and COC-Behav groups) revealed no effects of cocaine on c-Fos expression in the pyramidal cell layers (Table S1).

### Cocaine-withdrawn mice show altered regulation of DG GABAergic neurons and adult hippocampal neurogenesis

**Fig. 5.**
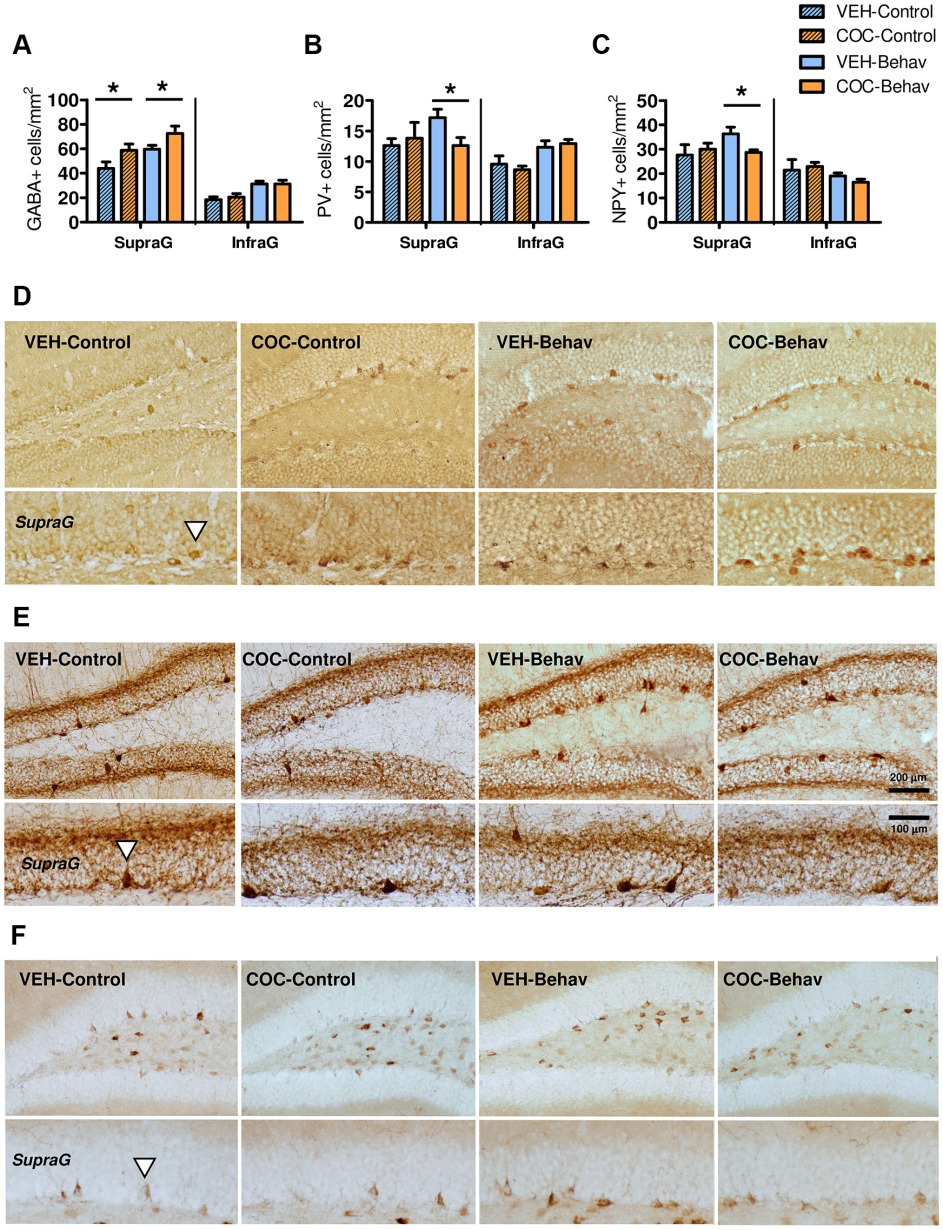
**Chronic cocaine withdrawal dysregulates GABAergic neuron populations in the DG.** All groups of cocaine-withdrawn mice showed increased numbers of GABA^+^ cells in the supragranular DG (A,D; and -Basal mice in Fig. S1). There was no difference in the populations of PV^+^ and NPY^+^ neurons, the COC- and VEH-treated mice when they were not submitted to behavioral stimulation (B,C,E,F; Fig. S1). However, after behavioral training, the cocaine-withdrawn mice (COC-Behav) showed a reduced number of PV^+^ and NPY^+^ neurons in the supragranular DG compared with VEH-Behav mice (B,C,E,F). Results are represented as the mean number of positive cells per mm^2^±s.e.m. Arrowheads indicate positive cells. Scale bars in E also apply for D and F. *Post hoc* LSD test: *P<0.05 for VEH versus COC.

Similar to the c-Fos result, a persistent increase in the number of GABA^+^ neurons was found in the DG in all the cocaine-withdrawn groups (‘DG blade’: *F*_1,28_=11.651, *P*=0.002; ‘treatment×DG blade’: *F*_1,28_=9.830, *P*=0.004; LSD is shown in Fig. 5A,D; this effect was replicated in the -Basal mice shown in Fig. S1). Behavioral training increased the number of GABA^+^ neurons in the DG, but this enhancement was not modulated by cocaine withdrawal, as it was similar in both VEH- and COC-Behav mice (‘behavior’: *F*_1,28_=11.651, *P*=0.002 in Fig. 5A,D; and analysis in Fig. 7B).

**Fig. 6.**
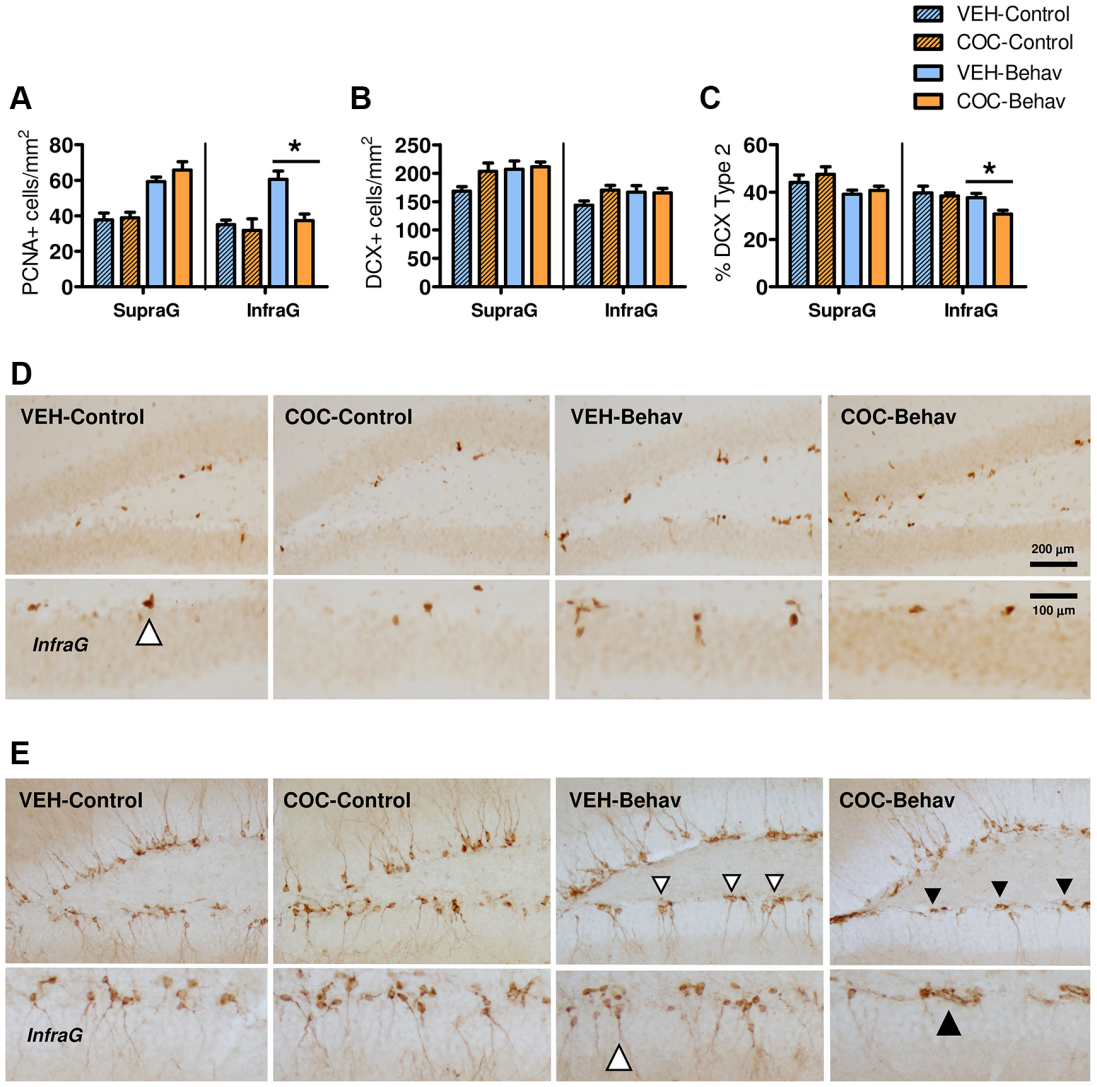
**Effect of chronic cocaine withdrawal on adult hippocampal neurogenesis.** Vehicle- and cocaine-treated mice (VEH- and COC-) showed no differences in AHN-related parameters when evaluated in control conditions (A-E; Fig. S1). However, after behavioral training, the COC-Behav mice showed a reduced PCNA expression in the infragranular blade (A,D) and a reduced percentage of mature-like ‘Type 2’ DCX^+^ neurons (C,E) in this region. Results are represented as means±s.e.m. Arrowhead in D indicates positive cell. In E, the white arrowheads indicate DCX^+^ ‘Type 2’ mature-like neurons, whereas black arrowheads indicate DCX^+^ immature-like ‘Type 1’ neurons. Scale bars in D also apply to E. *Post hoc* LSD test: *P<0.05 for VEH versus COC.

However, withdrawal from chronic cocaine did not induce long-lasting alterations in the number of PV^+^ and NPY^+^ interneuron populations in the DG (Fig. 5B,C) or AHN-related markers (proliferating PCNA^+^ cells and DCX^+^ immature neurons; Fig. 6), when these were assessed under ‘resting’ conditions. It was observed that the numbers were similar in the VEH-Control and the COC-Control mice, and results in the -Basal groups also revealed no differences (Fig. S1). The behavioral training notably increased hippocampal plasticity by enhancing the expression of both PV^+^ and NPY^+^ neurons (ANOVA effects for PV: ‘behavior’: *F*_1,28_=6.080, *P*=0.020; ‘DG blade’: *F*_1,28_=17.804, *P*=0.000; ‘treatment×behavior×DG blade’: *F*_1,28_=4.177, *P*=0.050; ANOVA effects for NPY: ‘DG blade’: *F*_1,28_=68.400, *P*=0.000; ‘DG blade×behavior’: *F*_1,28_=9.128, *P*=0.005; LSD is shown in Fig. 5B,C,E,F) and by modulating AHN-related markers (ANOVA effects for PCNA: ‘behavior’: *F*_1,28_*=*29.728, *P*=0.000; ‘DG blade’: *F*_1,28_=10.468, *P*=0.003; ‘DG blade×treatment’: *F*_1,28_=8.758, *P*=0.006; ‘treatment×behavior×DG blade’: *F*_1,28_=4.396, *P*=0.045; ANOVA effects for the % of mature-like ‘Type 2’ DCX: ‘behavior’: *F*_1,28_=9.935, *P*=0.004; ‘DG blade’: *F*_1,28_=111.00, *P*=0.000; ‘DG blade×treatment’: *F*_1,28_=20.007, *P*=0.000; ‘treatment×behavior×DG blade’: *F*_1,28_=5.128, *P*=0.031; LSD is shown in Fig. 6A,C,D,E). Nonetheless, *post hoc* comparisons revealed differences between the VEH-Behav and the COC-Behav mice, suggesting that they underwent a different neuroplastic modulation after behavior in certain DG blades (Figs 5 and 6).

**Fig. 7.**
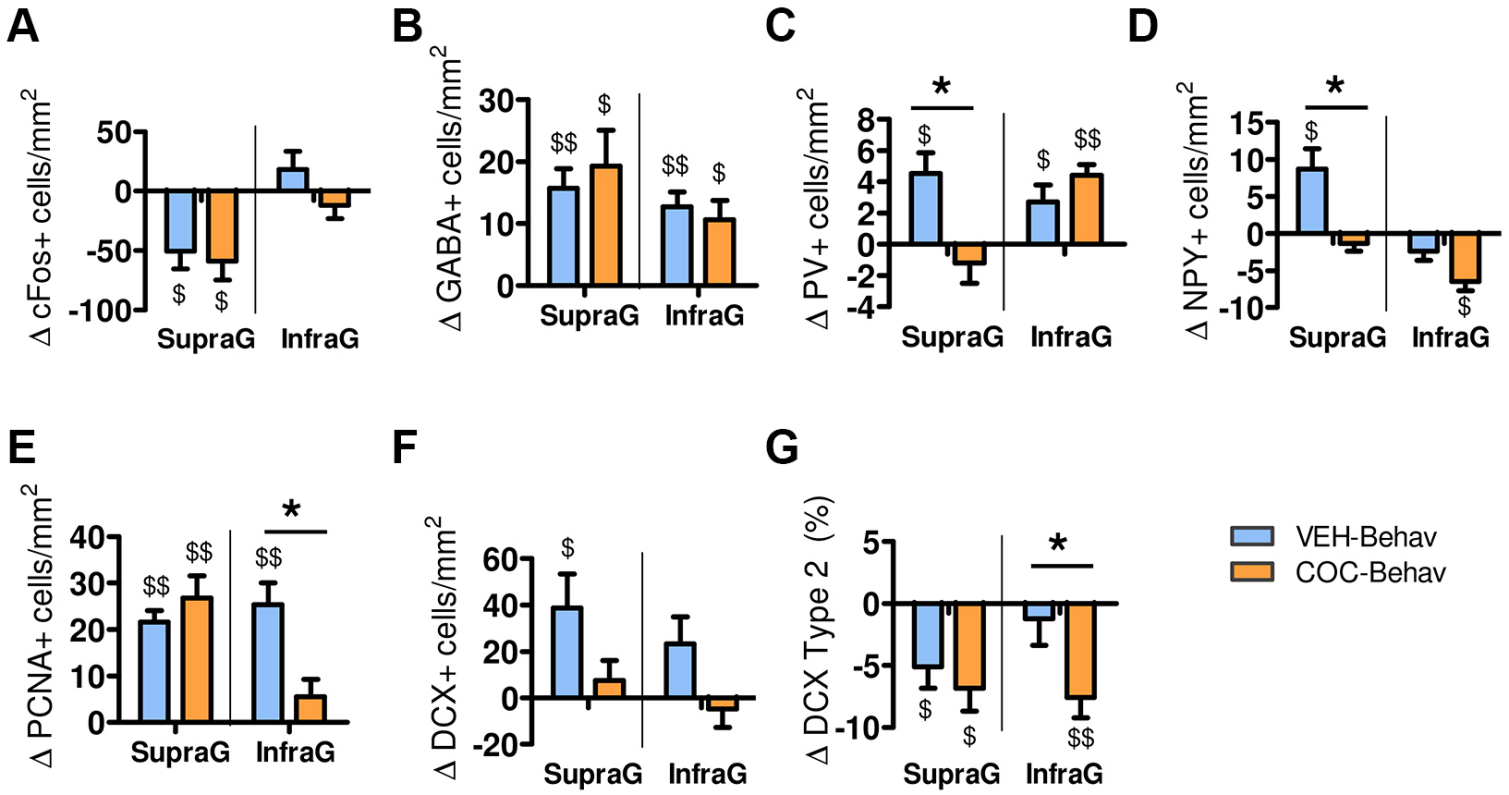
**Behavior-induced modulation of plasticity in the DG is altered in cocaine-withdrawn mice.** Change (Δ) in DG marker expression induced by behavior in VEH-Behav and COC-Behav mice compared with their respective control group (represented by zero in the graphs). The VEH-Behav mice showed notable behavior-induced changes, reducing basal c-Fos expression (A) but increasing GABAergic neuron populations (B-D) and adult hippocampal neurogenesis (E,F) in the DG after behavior training. However, the behavior-induced upregulation of PV and NPY was blunted in the supragranular layer of the COC-Behav mice (B,C). (G) In addition, the COC-Behav mice showed a blunted regulation of PCNA (E) and a reduced maturation of the young DCX^+^ neurons in the infragranular blade. Results are represented as mean±s.e.m. difference from the respective control group. *Post hoc* LSD test: *P<0.05 for VEH versus COC. One-sample Student's *t*-tests to compare means versus zero: ^$^*P*<0.05, ^$$^*P*<0.001 (this comparison indicates a significant change from the respective control group).

Therefore, the impact of the behavioral training on the DG during cocaine withdrawal was further explored by analysing the relative change found in the -Behav mice groups from their respective -Control group (supplementary Materials and Methods). Results confirmed that the COC-Behav mice showed a reduction in both PV^+^ and NPY^+^ cells in the supragranular region [ANOVA effects: for PV: ‘treatment×DG blade’: *F*_1,19_=12.114, *P*=0.003; ANOVA effects for NPY: ‘treatment’: *F*(1, 19)=10.176, *P*=0.005; ‘DG blade’: *F*_1,19_=39.062, *P*=0.000; ‘treatment×DG blade’: *F*_1,19_=8.073, *P*=0.010; LSD is shown in Fig. 7C,D]. In addition, the COC-Behav mice dysregulated their expression of PCNA (‘DG blade’: *F*_1,19_=5.064, *P*=0.036; ‘treatment×DG blade’: *F*_1,19_=11.692, *P*=0.003; Fig. 7E) and DCX in the infragranular layer. They showed a reduced level of cell proliferation, but this did not affect the total number of young neurons. However, there was a reduction in the frequency of ‘Type 2’ mature-like morphology cells (‘treatment×DG blade’: *F*_1,19_=5.474, *P*=0.030; LSD is shown in Fig. 7F,G).

Finally, it is worth mentioning that the VEH-Behav and COC-Behav groups showed no remarkable differences in their GABAergic neuron populations assessed in the CA1 and CA3 hippocampal regions (Table S1) or in the turnover of the adult-born cells assessed by the amount of apoptotic nuclei in the granular and subgranular zones (Fig. S2).

## DISCUSSION

This study describes long-lasting memory deficits in mice withdrawn from cocaine, that show persistent neuroadaptations in the hippocampal DG concerning increased basal neuronal activity as well as an altered regulation of the GABAergic neurons and AHN after behavioral training. This pre-clinical evidence will help to characterize the neurobiological basis of the hippocampal-dependent emotional and cognitive alterations occurring during cocaine withdrawal.

Mice were submitted to a chronic cocaine treatment for 12 days and then to 44 days of a drug-free period before the behavioral assessment started. The cocaine-withdrawn mice displayed normal exploratory behavior across testing, and their emotional alterations were subtle since only the plus maze test (which probably elicited the most anxiogenic situation considering that it was performed before mice were habituated to the testing procedure) revealed a tendency to increased anxiety in the cocaine-treated animals. These results apparently disagree with previous works indicating that both acute and chronic cocaine exposure have clear anxiogenic properties (Paine et al., 2002; Yang et al., 1992) and that drug withdrawal is characterized by a high-anxiety state that may occur alongside defenseless behaviors in rodents (Alves et al., 2014; El Hage et al., 2012; Erb et al., 2006; Harris and Aston-Jones, 1993; Perrine et al., 2008; Santucci and Rosario, 2010; Sarnyai et al., 1995; Valzachi et al., 2013). Nonetheless, it should be highlighted that the aforementioned experiments used cocaine withdrawal periods significantly shorter (24 h to 28 days) than our experimental protocol (44 days), suggesting that anxiety may gradually decline after prolonged cocaine abstinence.

By contrast, the cocaine-withdrawn mice displayed notable cognitive deficits as reflected by impaired reference memory (24 h recognition of familiar objects and locations), impaired acquisition of drug-contextual associations (CPP test) and impaired spatial working memory (continuous spontaneous alternation in the Y-maze). It should be noted that this study does not provide direct evidence on the degree of hippocampal participation in the aforementioned tasks. In this regard, the hippocampal involvement in object recognition memory is controversial, as it may depend on several aspects in the testing protocol [such as complexity of the testing environment (Denny et al., 2012) or the temporal delay between the sample and test sessions (Cohen and Stackman, 2015)]. Nevertheless, the literature reports a clear role of the hippocampus for both the place recognition memory (Barker and Warburton, 2011; Broadbent et al., 2004) and the acquisition and processing of cocaine–stimuli associations in the CPP paradigm (Castilla-Ortega et al., 2016b; Hernández-Rabaza et al., 2008). Thus, our results strongly support a defective hippocampal-dependent memory in the cocaine-withdrawn mice.

Hippocampal-dependent memory impairment has been reported in rodents withdrawn from chronic cocaine (Briand et al., 2008; Burke et al., 2006; Krueger et al., 2009) even after a long withdrawal period of 3 months (Mendez et al., 2008). A novelty of this study is that multiple tasks were performed to evaluate both emotion and cognition. The results suggest that the cocaine-induced cognitive impairment is independent of the emotional symptoms, and this alteration may persist over time even after recovery at the emotional level. Also, because emotion and memory are supported by different neurobiological mechanisms and are dissociated within the hippocampus (Bannerman et al., 2004; Bertoglio et al., 2006), it is possible to explain the different performances in these two domains.

Hippocampal histological markers were evaluated at day 73 of cocaine withdrawal. A main finding is that the cocaine-withdrawn mice, regardless of whether they were submitted to behavioral training or not, displayed enhanced basal c-Fos activity in the hippocampus, specifically located in the DG. This shows persistent neuroadaptations increasing DG basal excitatory tone during cocaine withdrawal, which probably impairs any further response of the hippocampus to environmental/behavioral demands, since hippocampal neuronal activity supports the encoding and consolidation of stimuli into memories (Kheirbek et al., 2013). The enhanced basal c-Fos activity paralleled the up-regulation of the GABA^+^ neurons in the DG region, suggesting that compensatory mechanisms attempt to enhance the inhibition. However, the expression of markers for specific GABAergic interneuron populations, such as PV and NPY, was not increased in the cocaine-withdrawn animals. A possible explanation for this discrepancy is that the enhanced GABA^+^ cell population in the cocaine-withdrawn DG not only comprises interneuron subtypes such as those expressing PV and NPY, but excitatory granular projection neurons that modify their neurochemical content (Kahn et al., 2005). It has been widely reported that glutamatergic DG neurons constitutively contain both GABA and its synthesizing enzymes, and they may rapidly change to a GABAergic phenotype in pathological conditions of hippocampal hyper-excitation (Makiura et al., 1999; Ramírez and Gutiérrez, 2001; Sloviter et al., 1996, 2003), as may happen after cocaine withdrawal. Interestingly, an increment of GABA-synthesizing neurons in the DG has been described in mice abstinent from other dependence-inducing drugs (morphine) (Kahn et al., 2005). To the best of our knowledge, few previous studies have investigated the impact of cocaine on the hippocampal GABAergic neurons, and none tested the animals after long withdrawal periods. The available evidence (Goodman and Sloviter, 1993) is in concordance with our result as it showed that a repeated high cocaine dosage does not apparently induce neurotoxicity, nor does it alter basal expression of GABAergic interneuron populations markers in the hippocampus (numbers of PV^+^, calretinin^+^ and somatostatin^+^ neurons were unchanged; changes were found in NPY immunoreactivity but these were elicited by cocaine-induced seizures and not by cocaine administration itself).

Thus, under non-stimulating conditions, the cocaine-withdrawn mice showed no evident alterations in the expression of PV and NPY in the DG; and their AHN-related markers (cell proliferation and DCX^+^ neurons) were also normal. In contrast, these populations were significantly altered in the cocaine-withdrawn mice submitted to behavioral training. The behavioral training used in this study included a number of hippocampal-relevant stimuli, such as exposure to novel contexts and objects, locomotion/exercise opportunities, mild anxiogenic experiences (i.e. unknown environments and inescapable situations) and engagement in learning activities. Thus, while it is not possible to link a particular stimulus or behavior to its corresponding neurobiological correlate, the whole behavioral training protocol could be understood as an ‘enriched’ or ‘hippocampus-demanding’ environment that may have an impact on the hippocampus. Both AHN and the hippocampal PV^+^ and NPY^+^ populations have been demonstrated to increase their expression in response to exercise (Arida et al., 2004; Bjørnebekk et al., 2006; Nguyen et al., 2013; van Praag et al., 1999), environmental enrichment (Bjørnebekk et al., 2007; Iuvone et al., 1996; Kempermann et al., 1997; Suzuki et al., 2014) and/or hippocampal-dependent learning (Donato et al., 2013; Gould et al., 1999). Accordingly, our results showed that normal mice submitted to behavioral training potentiated inhibitory mechanisms in the DG, as reflected by a reduction in basal c-Fos activity with increased PV^+^, NPY^+^ and GABA^+^ neurons in the supragranular cell layer. This response was accompanied by a notable upregulation of cell proliferation in both DG blades and by an increase in the number of DCX^+^ neurons. On the contrary, the cocaine-treated mice exposed to behavior presented an alteration of this process. They did not upregulate their PV^+^ and NPY^+^ populations, and their proliferating response was blunted in the infragranular blade, where their DCX^+^ neurons showed abnormal morphological features suggesting a block or delay in maturation. An insufficient or aberrant neuroadaptive response of the DG to environmental stimuli may contribute to the cognitive symptoms induced by cocaine withdrawal. Both the hippocampal PV^+^ and NPY^+^ interneurons and AHN have a well-established role in the hippocampal-dependent emotional and cognitive processes (Beck and Pourié, 2013; Castilla-Ortega et al., 2011; Christiansen et al., 2014; Deng et al., 2010; Fuchs et al., 2007; Korotkova et al., 2010; Leuner et al., 2006; Morellini et al., 2010; Quarta et al., 2015; Zou et al., 2016) and, specifically, the experience-dependent remodeling of the hippocampal PV-related networks (Donato et al., 2013) and AHN (Castilla-Ortega et al., 2011; Deng et al., 2010; Leuner et al., 2006) are key neuroplastic processes for memory consolidation, retrieval and learning.

Cocaine exposure and withdrawal may notably modulate hippocampal activity and neuroplasticity since the hippocampus receives dopaminergic projections from a main reward center such as the ventral tegmental area (Castilla-Ortega et al., 2016b). Acute cocaine exposure enhances hippocampal early-immediate gene expression (Schöpf et al., 2015) and fMRI signal (Febo et al., 2005) but this response attenuates after repeated dosage, which indicates synaptic changes (Febo et al., 2005). The sculpting of the hippocampal circuitry by cocaine may involve a number of mechanisms: (1) it promotes the formation of dendritic spines in hippocampal neurons (Fole et al., 2011; Miguens et al., 2015); (2) it enhances hippocampal long-term potentiation (Gabach et al., 2013; Keralapurath et al., 2014), which, in turn, seems occluded after long cocaine access or extended cocaine withdrawal (Keralapurath et al., 2015; Thompson et al., 2004); (3) it dysregulates hippocampal neurotransmission [e.g. glutamate, GABA or cannabinoid signaling (Blanco et al., 2012, 2016; Li et al., 2003)]; (4) it hampers neurotrophic and inflammatory factors (Zhu et al., 2016); and (5) it temporally reduces AHN (Castilla-Ortega et al., 2016b). Accordingly, cocaine addicts show profound neurobiological alterations in the hippocampus, as evidenced by *in vivo* studies of hippocampal functional activity and connectivity [both in basal/resting conditions and after stimulation (Adinoff et al., 2015; Castilla-Ortega et al., 2016b; Ding and Lee, 2013)] and by *post mortem* gene expression analysis (Enoch et al., 2014, 2012; Mash et al., 2007; Zhou et al., 2011). Because the hippocampus reciprocally projects in the reward areas, it is part of the ‘cocaine addiction circuit’ (Castilla-Ortega et al., 2016b), where altered hippocampal activity after cocaine exposure contributes, in turn, to maintain cocaine-related behaviors. In this regard, as revealed by clinical and/or pre-clinical experiments, the hippocampus is involved in the acquisition and engrained retention of drug-contextual associations (Fuchs et al., 2005; Hernández-Rabaza et al., 2008; Otis et al., 2014), sensitization to the stimulant locomotor effects of cocaine (Blanco et al., 2016), which was shown by our cocaine-withdrawn mice when tested in the CPP, and the craving and relapse outcomes elicited by cocaine-associated stimuli (Kilts et al., 2004; Potenza et al., 2012; Tomasi et al., 2015). Although this aspect has been less well explored, the fact that both cocaine addicts and cocaine-withdrawn rodents fail in cognitive tasks that typically involve the hippocampus (e.g. in addicts: Aharonovich et al., 2006; Fox et al., 2009; Vonmoos et al., 2013, 2014; in rodents: Briand et al., 2008; Burke et al., 2006; Krueger et al., 2009; Mendez et al., 2008 and the present work) supports the idea that an aberrant hippocampal function also contributes to the cocaine-induced cognitive decline.

Profound cognitive deficits involving global cognitive impairment are present in ∼30% of cocaine addicts (and even in 12% of cocaine recreational users) and correlate with the amount of cocaine consumed (Vonmoos et al., 2013, 2014). During cocaine abstinence, cognitive damage may be recovered within a year (i.e. the involved neuroadaptations seem reversed or compensated) but only in those patients that completely cease from cocaine usage (Vonmoos et al., 2014). Furthermore, the presence of cognitive dysfunction in cocaine addicts is a strong predictor of relapse during the first months of drug withdrawal (Aharonovich et al., 2006; Fox et al., 2009; Teichner et al., 2002), supporting the importance of assessing and alleviating cognitive decline in cocaine addiction. This pre-clinical study shows that long-lasting cognitive deficits in mice withdrawn from cocaine are concomitant to (and, probably, at least partially explained by) hippocampal alterations involving increased DG neuronal activity, and an abnormal neuroplastic response of the interneuron populations and AHN to environmental demands (behavioral stimulation). These results emphasize the role of the hippocampus, and how this responds to external stimuli, as a key brain area to understand the long-term cocaine withdrawal manifestations. Future pre-clinical research should manipulate hippocampal inhibitory mechanisms and/or AHN to search for therapeutic approaches to treat the cocaine-induced cognitive symptoms, and to strengthen the link between these hippocampal processes and the behavioral effects of cocaine.

## MATERIALS AND METHODS

### Animals

Forty-four male C57BL/6J mice were acquired from Janvier Labs (Le Genest-Saint-Isle, France) at 8 weeks of age and underwent 1 week of acclimatization before the experiments started. Mice were housed in groups of 3-4 on a 12 h:12 h light/dark cycle in standard cages containing shredded paper as nesting material, water and food provided *ad libitum*. All experiments were performed in accordance with the European (Directive 2010/63/UE) and Spanish (Real Decreto 53/2013, Ley 32/2007 and 9/2003) regulations of animal research. The experimental protocols were approved by the research ethics committee of the University of Málaga (code: CEUMA 8-2014-A and 19-02-15-197).

### Cocaine treatment and experimental conditions

Mice were randomly assigned to a cocaine (‘COC’, *n*=22) or a vehicle (‘VEH’, *n*=22) condition. The ‘COC’ mice received a chronic cocaine treatment consisting of a daily intraperitoneal (i.p.) 20 mg/kg dose of cocaine (Sigma; diluted in 10 ml/kg volume of saline −0.9% NaCl) for 12 consecutive days; VEH mice received an equivalent i.p. saline volume. After the last cocaine or saline dose, all mice remained undisturbed for 44 days (Fig. 1). From days 45 to 62, both groups were split in the ‘-Behav’ mice (Fig. 1A; COC-Behav, *n*=11; VEH-Behav, *n*=10) that were submitted to a behavioral test battery to evaluate hippocampal dependent behavior, including training in a cocaine-induced CPP paradigm; and the ‘-Control’ mice (Fig. 1B; COC-Control, *n*=5; VEH-Control, *n*=6) that were not exposed to any behavioral training but received home-cage cocaine injections (2.5 mg/kg/day on days 52-55; 10 mg/kg/day on days 57-60), as a control for the drug administration that the ‘-Behav’ mice received during their CPP training. Therefore, the -Behav and the -Control groups only differed in their exposure to training stimulation, so their comparison allows us to determine the impact of behavior on the histological parameters. The remaining mice (‘-Basal’) were included to assess the effects of the initial VEH- or COC- treatment in conditions of no further drug or behavioral stimulation. Thus, -Basal mice were exposed to home-cage vehicle injections during days 52-55 and 57-60 (Fig. 1C; VEH-Basal, *n*=6; COC-Basal, *n*=6). The experimental groups submitted to behavioral assessment used an increased sample size because the behavioral data are usually more variable than the histological results.

### Behavioral assessment

The behavioral tests (Fig. 1A) were performed on the basis of previously published protocols (Albasser et al., 2010; Castilla-Ortega et al., 2016a; Hughes, 2004; Ladrón de Guevara-Miranda et al., 2016; Santin et al., 2009) as detailed in the supplementary Materials and Methods. Briefly, for an evaluation of exploratory and anxiety-like behavior, mice underwent one session in the elevated plus maze (day 45 after the last vehicle or cocaine dose), in the light/dark box (day 46) and in the open field (day 47) tests. Cognitive function was subsequently assessed through a novel object recognition task (Fig. 3A) that measured memory for a familiar object (day 48) calculated as an ‘object memory ratio’ [(time exploring the novel object−time exploring the familiar object)/total time exploring both objects] and memory of a familiar place (day 49) calculated as a ‘place memory ratio’ [(time exploring the displaced object−time exploring the static object)/total time exploring both objects] (supplementary Materials and Methods). According to these ratios, a successful object or place memory would be indicated by a positive ratio score that is significantly different from zero (Albasser et al., 2010). Finally, depression-like behavior was evaluated in the forced swimming test (day 50). The rationale underlying this test order was to assess exploration and anxiety-like behavior first, when animals are less habituated to the environment so it would elicit more novelty and aversion, whereas a highly stressful task such as the forced swimming test was administered last to avoid its potential impact on later behavioral measures.

Cocaine-induced locomotion and the acquisition of drug-contextual associations were subsequently evaluated in a CPP paradigm. In one habituation session (day 51), the behavior-training mice freely explored a CPP apparatus that consisted of two compartments distinguishable by the contextual cues decorating their walls and communicated by a clear corridor. The time spent in each compartment was recorded and the compartment least preferred by a mouse was paired with a cocaine injection in the conditioning phase, while the other compartment was paired with a saline injection. To study the effects of both a low and a high cocaine dose, conditioning was first carried out with a dose of 2.5 mg/kg/day (days 52-55) of cocaine followed by a test session (Test 1; day 56); and then with a dose of 10 mg/kg/day of cocaine (days 57-60), followed by a second test session (Test 2; day 61). Test sessions consisted of free exploration of the CPP apparatus after saline administration. A ‘CPP ratio’ [(seconds spent in the cocaine-paired compartment−seconds spent in the saline-paired compartment)/total seconds spent in both compartments]×100 was calculated, so the preference for the cocaine-paired compartment over the saline-paired one would be indicated by a positive CPP ratio, significantly different from zero (Castilla-Ortega et al., 2016a; Ladrón de Guevara-Miranda et al., 2016; Poltyrev and Yaka, 2013).

The continuous spontaneous alternation in the Y-maze was evaluated at the end of the testing (day 62). An ‘alternation score’ [number of spontaneous alternations/(total number of arm entries−2)] (Hughes, 2004) was calculated for the first 30 possible alternations (i.e. 32 arm entries) performed.

### Immunohistochemistry and cell quantification

On day 73, mice from all experimental conditions were deeply anesthetized for transcardial perfusion with 0.1 M phosphate-buffered saline pH 7.4 (PBS). Their brains were dissected out and cut through the midline. The left hemisphere was arbitrarily chosen for the histological study. It was post-fixed for 48 h in 4% paraformaldehyde in PBS and cut into coronal vibratome sections (50 µm) from −1.06 to −3.08 mm from bregma (Paxinos and Franklin, 2001), which includes the hippocampal area. For free-floating immunohistochemistry, sections first received a heat-induced epitope retrieval (EnVision Flex high pH solution; Dako, Glostrup, Denmark) followed by an endogenous peroxidase blocking (80% PBS, 10% methanol and 10% hydrogen peroxidase) for 30 min in the dark and incubated overnight with the corresponding primary antibody. A mouse anti-c-Fos antibody (1:500, Santa Cruz Biotechnology, sc-271243, lot 10413; which is also reactive for the c-Fos functional homologs Fos B, Fra-1 and Fra-2) was used for detection of basal neuronal activity; while the GABAergic interneuron populations were assessed by rabbit anti-PV (1:500; Swant, Marly, Switzerland, PV-28, lot 5.10), rabbit anti-NPY (1:500, Sigma, N9528, lot R40829) and rabbit anti-GABA (1:500, Sigma, A2052, lot 095K4830) antibodies. The AHN-related markers used were a mouse anti-proliferating cell nuclear antigen (PCNA; 1:1000, Sigma, P8825, lot 014M4836) to label cells undergoing proliferation in the dentate gyrus, and goat anti-doublecortin (DCX; 1:200, Santa Cruz, sc-8066, lot A1211) for immature neurons up to 3-4 weeks of age (Brown et al., 2003). All antibodies were diluted in PBS, 0.5% Triton X-100 and 2.5% donkey serum. On the second day, sections were incubated for 90 min in a biotin-conjugated secondary antibody (rabbit anti-mouse, rabbit anti-goat or swine anti-rabbit as appropriate; Dako; diluted 1:800) and for 1 h in peroxidase-conjugated extravidin (Sigma, 1:1000 in PBS) solution in the dark. The staining solution contained diaminobenzidine (DAB) as the chromogen (proportion: 0.1 ml of DAB previously diluted at 5% in distilled water, 10 µl hydrogen peroxidase and 10 ml PBS) and nickel chloride (NiCl_2_, Sigma) was added to intensify c-Fos labelling (0.004 g per 10 ml of staining solution). Each step was followed by PBS rinses. Negative controls in which the primary antibody was omitted resulted in absent staining.

Markers were quantified separately in the supragranular and infragranular cell layers of the DG in all mice, while other DG regions and the cornu ammonis (CA) were further explored in the -Behav mice groups. Importantly, it has been recently emphasized that the supragranular and infragranular blades should be considered as different functional and morphological divisions within the DG (Gallitano et al., 2016; Jinno, 2011). Cell counting was carried out following our previously reported methods (Castilla-Ortega et al., 2016a, 2013, 2014). Cells were quantified in 1 of every 8 hippocampal sections for each marker in high-magnification photographs (4080×3072 pixels, using a 10× lens for the DG or a 4× lens for the CA) captured with an Olympus BX51 microscope equipped with an Olympus DP70 digital camera (Olympus, Glostrup, Denmark). The boundaries of the different hippocampal regions were defined according to anatomical criteria (DG: molecular, supragranular, infragranular and polymorphic layers; CA1: stratum oriens, pyramidal, radiatum and lacunosum; CA3: stratum oriens, pyramidal and radiatum) (Paxinos and Franklin, 2001). Using ImageJ (National Institutes of Health, Maryland, USA), the area of each region of interest was drawn, measured, and the positive cells within the region were manually counted. Data for each animal were expressed as the average number of positive cells per unit area (mm^2^). In addition, the DCX^+^ cells were classified into two cell types: ‘Type 1’: these were cells with absent or short dendritic processes (a morphology that usually corresponds to the most immature DCX+ cells) or ‘Type 2’: which were mature-like cells with at least one prominent apical dendrite penetrating the granule cell layer (Castilla-Ortega et al., 2016a, 2014; Plümpe et al., 2006).

### Statistical analysis

Comparisons among groups were carried out with Student's *t*-tests; or with repeated-measures analysis of variance (ANOVA) followed by *post hoc* Fisher's LSD analysis for between-groups comparisons when appropriate (Tybout et al., 2001). A one-sample *t*-test was used to compare means to a single measure. Significance was considered at *P*≤0.05. Only significant comparisons are reported.

To simplify and reduce the cognitive-related behavioral data, a principal component factorial analysis was performed as described in the supplementary Materials and Methods. In addition, to confirm whether behavioral training differently modulated hippocampal neuroplasticity in the cocaine-withdrawn mice, expression data from each histological marker in the -Behav mice was transformed to its relative change (Δ) from the respective -Control group (supplementary Materials and Methods).
